# Mutations in Homocysteine Metabolism Genes Increase Keratin *N*-Homocysteinylation and Damage in Mice

**DOI:** 10.1155/2018/7570850

**Published:** 2018-09-23

**Authors:** Kamila Borowczyk, Jacek Wróblewski, Joanna Suliburska, Noriyuki Akahoshi, Isao Ishii, Hieronim Jakubowski

**Affiliations:** ^1^Department of Microbiology, Biochemistry and Molecular Genetics, Rutgers-New Jersey Medical School, International Center for Public Health, Newark, NJ 07103, USA; ^2^Department of Environmental Chemistry, Faculty of Chemistry, University of Łódź, 90-236 Łódź, Poland; ^3^Institute of Bioorganic Chemistry, 71-704 Poznań, Poland; ^4^Department of Biochemistry and Biotechnology, Poznań University of Life Sciences, 60-632 Poznań, Poland; ^5^Institute of Human Nutrition and Dietetics, Poznań University of Life Sciences, 60-632 Poznań, Poland; ^6^Department of Health Chemistry, Showa Pharmaceutical University, Tokyo 194-8543, Japan

## Abstract

Genetic or nutritional deficiencies in homocysteine (Hcy) metabolism increase Hcy-thiolactone, which causes protein damage by forming isopetide bonds with lysine residues, generating *N*-Hcy-protein. In the present work, we studied the prevalence and genetic determinants of keratin damage caused by homocysteinylation. We found that in mammals and birds, 35 to 98% of Hcy was bound to hair keratin via amide or isopeptide bond (Hcy-keratin), while 2 to 65% was *S*-Hcy-keratin. A major fraction of hair Hcy-keratin (56% to 93%), significantly higher in birds than in mammals, was sodium dodecyl sulfate-insoluble. Genetic hyperhomocysteinemia significantly increased *N*-Hcy-keratin levels in the mouse pelage. *N*-Hcy-keratin was elevated 3.5-, 6.3-, and 11.7-fold in hair from *Mthfr*^−/−^, *Cse*^−/−^, or *Cbs*^−/−^ mice, respectively. The accumulation of *N*-Hcy in hair keratin led to a progressive reduction of *N*-Hcy-keratin solubility in sodium dodecyl sulfate, from 0.39 ± 0.04 in wild-type mice to 0.19 ± 0.03, 0.14 ± 0.01, and 0.07 ± 0.03 in *Mthfr*^−/−^, *Cse*^−/−^, or *Cbs*^−/−^animals, respectively. *N*-Hcy-keratin accelerated aggregation of unmodified keratin in *Cbs*^−/−^ mouse hair. Keratin methionine, copper, and iron levels in mouse hair were not affected by hyperhomocysteinemia. These findings provide evidence that pelage keratin is *N*-homocysteinylated *in vivo* in mammals and birds, and that this process causes keratin damage, manifested by a reduced solubility.

## 1. Introduction

Homocysteine (Hcy) is an important intermediate in folate and one-carbon metabolism. The only known source of Hcy in our body is the essential dietary protein amino acid methionine (Met). Hcy levels are regulated by vitamin B_12_- and folate-dependent remethylation to Met, catalyzed by Met synthase (with methyltetrahydrofolate cofactor provided by the MTHFR enzyme) and betaine-Hcy methyltransferase, as well as by vitamin B_6_-dependent transsulfuration to cysteine, the first step of which is catalyzed by cystathionine *β*-synthase (CBS). Genetic or nutritional deficiencies in folate/one-carbon metabolism lead to the hyperhomocysteinemia (HHcy) and are known to cause abnormalities in many organs, including the cardiovascular system and the brain [1].

Hcy is also metabolized to the thioester Hcy-thiolactone in an error-editing reaction in protein biosynthesis when Hcy is erroneously selected in place of Met by methionyl-tRNA synthetase [2]. Because Hcy-thiolactone is chemically reactive, it modifies *ε*-amino groups of protein lysine residues, which generates *N*-homocysteinylated protein (*N*-Hcy-protein) [2]. *N*-Homocysteinylation is an emerging posttranslational protein modification [3] that impairs or alters the protein's structure/function, causes protein damage *in vitro* [2, 4] and *in vivo* [5–7], generates amyloid-like structures [2, 8, 9], and induces proatherogenic changes in gene expression [10], an autoimmune response [2, 11], and atherothrombosis [2, 12, 13].

Although homocysteinylation can cause protein damage, the prevalence and genetic determinants of Hcy-damaged proteins *in vivo* are not well known. For this reason, the present work has been undertaken to study hair keratin from different species of mammals and birds, as well as mouse models of genetic HHcy (*Cbs*^−/−^, *Cse*^−/−^, and *Mthfr*^−/−^ mice).

## 2. Materials and Methods

### 2.1. Reagents

*L*-Hcy-thiolactone·HCl, *D*,*L*-Hcy, horse spleen ferritin, dithiothreitol (DTT), *o*-phthaldialdehyde (OPA), sodium dodecyl sulfate (SDS), HCl, and trichloroacetic acid were purchased from Sigma-Aldrich. Suprapure nitric acid (65%) and perchloric acid (60%) were from Merck. Reagents were prepared in Milli-Q purified water.

### 2.2. Animal Hair

The hair samples have been collected from live domestic animals and from wild animals encountered in nature as a fresh road kill. Random feathers dropped by wild birds were collected in nature. Woolly mammoth hair was purchased from Educational Innovations.

Cystathionine *β*-synthase-deficient (*Cbs*^−/−^) [14] and cystathionine *γ*-lyase-deficient (*Cse*^−/−^) [15] mice on C57BL/6J background, methyltetrahydrofolate reductase-deficient (*Mthfr*^−/−^) mice on a mixed BALB/cAnNCrlBR [16], and the corresponding wild-type control animals, 9- to 12-week-old, were used as hair donors. Hair samples were sheared off from the mouse sides.

### 2.3. Sample Preparation for Hair *S*-Hcy-Keratin and Hcy-Keratin Assays

Samples were prepared by a modification of previously described procedures as described below. The interassay and intra-assay variabilities for the quantification of various forms of Hcy were 7.3 and 11.5% [17].

### 2.4. *S*-Hcy-Keratin

Hair or feathers (2 mg) were treated in a 0.5 ml Eppendorf polyethylene tube with 50 mM Na_2_HPO_4_, 20 mM NaOH, 25mM DTT, and 1% SDS (200 *μ*l) for 1 h at 65°C. The extracts were collected by centrifugation, hair were extracted again with a fresh solution, and the extracts were combined (400 *μ*l). The hair pellets were saved for quantification of SDS-insoluble *N*-Hcy-keratin.

A 40 *μ*l portion of each extract was treated with DTT (4 *μ*l, 0.25M) and HCl (4 *μ*l, 12 N) at 100°C for 30 min to convert the liberated Hcy to Hcy-thiolactone, which was then quantified by HPLC [17]. Authentic samples of Hcy were similarly processed as standards for *S*-Hcy assays. This procedure liberates >95% Hcy present in hair as *S*-Hcy-keratin.

### 2.5. SDS-Soluble and SDS-Insoluble Hcy-Keratin

For SDS-soluble Hcy-keratin quantification, each SDS extract (300 *μ*l) was supplemented with 34 *μ*l 100% trichloroacetic acid and the precipitated keratin pellets were collected by centrifugation. SDS-soluble and SDS-insoluble pellets were transferred to 1 ml Wheaton Gold Band ampoules and hydrolyzed with 6N HCl and 50 mM DTT (110 *μ*l, 120°C, 1 h) to liberate Hcy. The hydrolysates were lyophilized on Labconco CentriVap (40 min, 70°C), dissolved in 10 *μ*l water, purified by two-dimensional TLC, and analyzed by HPLC. Horse spleen ferritin, containing 0.49 mol Hcy/mol protein, was processed in parallel as a standard. In these procedures, *N*-Hcy is quantitatively converted to Hcy-thiolactone, which is then assayed by HPLC.

### 2.6. HPLC, Detection, and Quantification

Quantification of Hcy-thiolactone generated from Hcy-keratin or *S*-Hcy-keratin was carried out as previously described [17] using a Beckman-Coulter System Gold Nouveau HPLC instrumentation with a manual injector (7725i Rheodyne, with 0.1 ml loop) and a Jasco 1520 fluorescence detector, controlled by a Gold Nouveau chromatography workstation software for Windows. Samples were injected onto a cation-exchange polysulfoethyl aspartamide column (35 × 2 mm, 5 *μ*m, 300 Å) (PolyLC Inc.), eluted isocratically with 30 mM NaCl and 10 mM sodium phosphate buffer (pH 6.6) at a flow rate 0.6 ml/min. The effluent was mixed in a three-way tee with 2.5 mM OPA and 0.25 M NaOH, delivered at a flow rate 0.3 ml/min, the mixture passed through a reaction coil (Teflon tubing, 0.3 mm I.D. × 3 m), and the fluorescence at 480 nm was recorded (excitation 370 nm). Hcy-thiolactone eluted at 3 min in a 4 min run.

### 2.7. Met-Keratin Assays

The Met HPLC assay [18] has been used with the following modification. Briefly, mouse pelage hair (~10 mg) were hydrolyzed in 1 ml Wheaton Gold Band ampoules with 6N HCl (100 *μ*l, 120°C, 1 h). The hydrolysates were dried out, dissolved in 50 *μ*l 0.2 M sodium phosphate buffer (pH 7.4), reduced with 2 *μ*l 0.25 M TCEP for 10 min, and supplemented with 10 *μ*l 0.5 M NAC. A Hewlett-Packard (Waldbronn, Germany) 1100 Series System, controlled by the HP ChemStation software, containing quaternary pump, autosampler, temperature control, vacuum degasser, and 1260 Series FL detector was used. Samples were analyzed using a reversed phase PRP-1 column (150 × 4.6 mm, 5 *μ*m; Hamilton, Energy Way, Reno, NV, USA) eluted at a flow rate 1 ml/min, 25°C, with 0.01 M OPA and 0.1 M NaOH (A) and acetonitrile (B) as follows: 0–8 min, 14–25% (B); 8–12 min, 25% (B); 12–14 min, 25–14% (B). The effluent was monitored by fluorescence (exc. 348 nm, em. 438 nm) from 0 to 7.2 min. Met was identified by coelution (at 6.2 min) with an authentic standard.

### 2.8. Total SDS-Soluble Keratin

Total SDS-soluble keratin was prepared by extracting mouse hair (2 mg) twice with 50 mM Na_2_HPO_4_, 20 mM NaOH, 25 mM DTT, and 1% SDS (200 *μ*l, 65°C, 1 h each). The extracts were collected by centrifugation and combined (400 *μ*l). A 20 *μ*l portion of each extract was analyzed by SDS-PAGE on 10% gels. Keratin bands were visualized by staining with Coomassie Blue and quantified by densitometry.

### 2.9. Copper and Iron Assays

Mouse hair samples were mineralized with 65% nitric acid (Merck) in a microwave oven (Mars 5 Digestion Microwave System, CEM Corporation). Iron and copper were quantified by flame atomic absorption spectrometry using a Zeiss AAS-3 spectrometer with deuterium background correction as previously described [19]. The accuracy of the assay was 94% for iron and 102% for copper as verified by certified reference materials (Human Hair NCS DC73347a, LGC Standards).

### 2.10. Statistics

The results are reported as mean ± standard deviation. Comparisons between two groups are analyzed by using two-sided Student's *t*-test. Relationships of Hcy-keratin solubility vs. levels were fitted to logarithmic equations and analyzed by linear regression. The level of statistical significance was set to *P* < 0.05.

## 3. Results

### 3.1. Hcy-Keratin and *S*-Hcy-Keratin Content in Animal Hair

Our previous work has shown that Hcy, bound by a disulfide or an amide bond, is a component of human hair keratin [18]. To determine whether the presence of Hcy in hair keratin is a universal feature conserved across the animal world, we analyzed Hcy content in pelages of mammals and birds. We found that both Hcy-keratin and *S*-Hcy-keratin were present both in mammal and bird pelages at levels ranging from 56 to 733 pmol/mg hair (Table 1). The higher content of Hcy-keratin and lower content of *S*-Hcy-keratin at the tip of the horse tail hair than at its base are similar to those observed previously in the long human head hair [18].

While an average Hcy-keratin level was similar in mammals and birds (277 ± 202 vs. 357 ± 244 pmol/mg hair, *P* = 0.518), significantly more *S*-Hcy-keratin was present in mammals than in birds (247 ± 146 vs. 82 ± 20 pmol/mg hair, *P* = 0.037), so that in birds, Hcy-keratin represented a significantly higher fraction of total keratin Hcy content than in mammals (0.79 ± 0.10 vs. 0.47 ± 0.15, *P* = 0.0003) (Table 2).

To determine whether the variation in keratin homocysteinylation across the mammalian and bird species can alter the protein structure, we measured the solubility of Hcy-keratin in sodium dodecyl sulfate (SDS) and examined how the solubility is affected by the level of homocysteinylation. We found that SDS-insoluble Hcy-keratin varied from 78 to 93% in birds and from 56 to 92% in mammals (Table 1). Overall, birds had significantly more SDS-insoluble Hcy-keratin than mammals (0.87 ± 0.07 vs. 0.75 ± 0.12, *P* = 0.019; Table 2).

### 3.2. Increased Keratin Homocysteinylation and Reduced Solubility in Hyperhomocysteinemia

HHcy is known to increase the generation of Hcy-thiolactone, which modifies protein lysine residues by *N*-homocysteinylation. To examine how *N*-homocysteinylation of keratin affects its structure *in vivo*, we used mouse models of HHcy. We found that genetic HHcy significantly increased the accumulation of Hcy-keratin and *S*-Hcy-keratin in mouse pelage. In hair from *Cbs*^−/−^, *Cse*^−/−^, or *Mthfr*^−/−^ mice, Hcy-keratin was elevated 11.7-, 6.3-, and 3.5-fold, respectively; *S*-Hcy-keratin was elevated 15-, 5.4-, or 2.0-fold, respectively (Table 3). We also found that dietary HHcy induced in wild-type mice by providing 1% Met in drinking water, significantly elevated *S*-Hcy-keratin levels (127 ± 10 vs. 303 ± 58 pmol/mg hair, *P* < 0.001) and had no significant effect on Hcy-keratin levels (87 ± 10 vs. 97 ± 11 pmol/mg hair) (Table 3).

**Figure 1:**
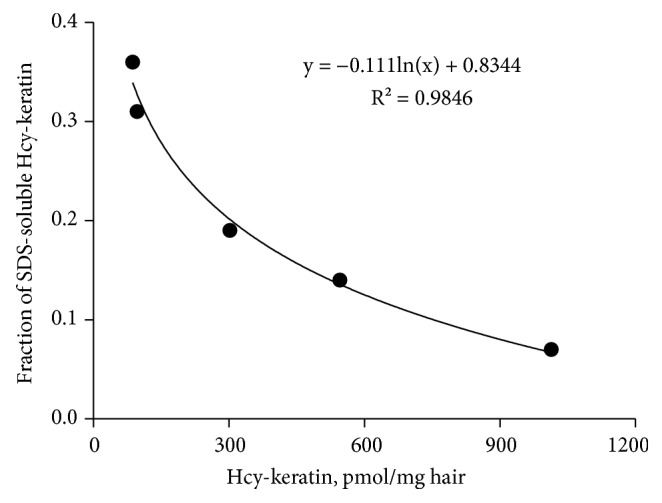
A relationship between the solubility of Hcy-keratin and the extent of keratin homocysteinylation. Mean values of Hcy-keratin solubility are plotted against mean Hcy-keratin levels (data from Table 3).

The accumulation of Hcy in keratin led to a significant reduction of its solubility in SDS, from 0.39 ± 0.04 in wild-type mice to 0.19 ± 0.03, 0.14 ± 0.01, and 0.07 ± 0.03 in *Mthfr*^−/−^, *Cse*^−/−^, and *Cbs*^−/−^animals, respectively (Table 3). There was a strong correlation between the solubility of *N*-Hcy-keratin in SDS and *N*-Hcy-keratin levels (Figure 1).

**Figure 2:**
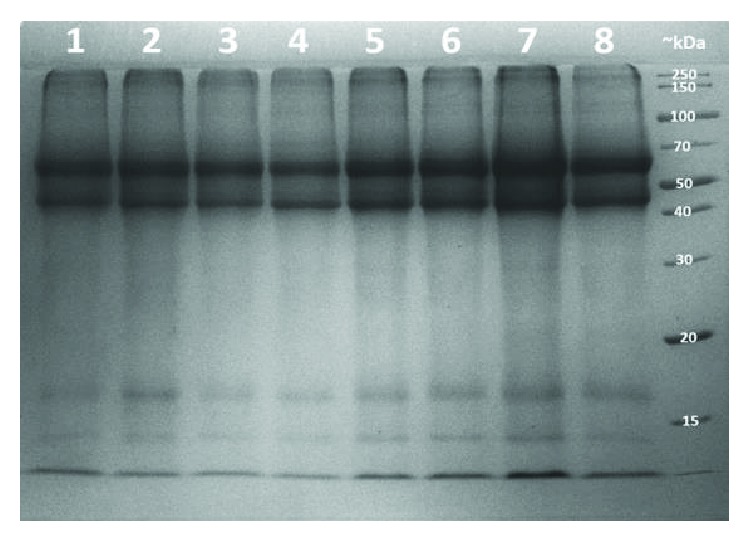
SDS-PAGE analysis of hair keratin from *Cbs*^−/−^ and *Cbs*^+/+^ mice. Keratin was extracted from mouse hair (stored at 25°C for 6 months) with SDS/DTT solution [18], and the extracts were analyzed on 10% SDS-PAGE gels. Lanes 1–4, keratin from *Cbs*^−/−^ mice; lanes 5–8, keratin from their *Cbs*^+/+^ littermates. Quantification by densitometry is shown in Table 4. The last lane on the right shows molecular weight markers.

SDS-PAGE analysis showed that similar amounts of SDS-soluble total keratin could be extracted from hair of *Cbs*^−/−^ mice and their *Cbs*^+/+^ littermates (Table 4). However, after a 6-month long storage of the hair at 25°C, significantly less of SDS-soluble total keratin was extracted from hair of *Cbs*^−/−^ mice than from hair of their *Cbs*^+/+^ littermates (1.51 ± 0.14 vs. 2.22 ± 0.05, *P* = 4.2 × 10^−6^) (Figure 2, Table 4).

### 3.3. Hair Copper, Iron, and Met-Keratin Are Not Affected by Hyperhomocysteinemia

In a growing human hair, Hcy-keratin levels increase along the hair shaft as a result of copper and iron-dependent demethylation of methionine residues of keratin [18]. To determine whether this process could contribute to the accumulation of Hcy-keratin in HHcy, we quantified copper and iron levels in hair as well as Met content of hair keratin in *Cse*^−/−^ and *Cse*^+/+^ mice. We found that there were no differences in copper (4.83 ± 0.70 vs. 5.37 ± 1.43 *μ*g/g hair, *P* = 0.296) and iron (34.6 ± 10.7 vs. 40.1 ± 16.0 *μ*g/g hair, *P* = 0.373) levels between *Cse*^−/−^ and *Cse*^+/+^ mice (Table 5). We also found that Met-keratin levels were not affected by the inactivation of the *Cse* gene (41.0 ± 10.2 in *Cse*^−/−^ vs. 38.8 ± 6.4 nmol/mg hair in *Cse*^+/+^ mice, *P* = 0.571) (Table 5).

## 4. Discussion

The present work shows that Hcy-keratin is highly prevalent in a variety of mammal and bird species. Although animal pelages contain both *S*-Hcy-keratin and Hcy-keratin, mammals have significantly more *S*-Hcy-keratin and less of the Hcy-keratin fraction than birds. At the same time, birds have significantly more SDS-insoluble Hcy-keratin than mammals, possibly suggesting higher keratin damage in birds than in mammals. We also show that the *Cbs*, *Cse*, and *Mthfr* genes control the extent of keratin *N*-homocysteinylation and damage in mice.

In hair, two mechanisms can account for the presence of Hcy linked by an amide bond to keratin: (i) *N*-homocysteinylation of keratin lysine residues by Hcy-thiolactone and (ii) copper- and iron-dependent demethylation of keratin methionine residues [18]. Although we have not directly shown that Hcy is attached to a lysine residue in keratin, we have eliminated the 2^nd^ possibility by showing that copper, iron, and keratin methionine levels are not affected by HHcy in the *Cse*^−/−^ mice (Table 5). In conjunction with previous findings that HHcy increases Hcy-thiolactone synthesis and protein *N*-homocysteinylation in mice and humans [2], our present data strongly suggest that the increased hair Hcy-keratin levels in HHcy mice are due to keratin *N*-homocysteinylation.

The higher content of Hcy-keratin and lower content of *S*-Hcy-keratin at the tip of the horse tail hair than at its base are reminiscent of those observed in the long human head hair [18]. Elevated Hcy-keratin at the tip of the horse tail hair relative to its base is most likely caused by demethylation of methionine residues in hair keratin, as we have previously shown for the human hair.

Previous work shows that *N*-homocysteinylation alters protein structure/function and leads to the generation of insoluble protein aggregates [20] with amyloid-like properties [2, 8]. The mechanism underlying these structural changes involves free Hcy thiol in *N*-Hcy-protein, which is prone to one-electron redox reactions that generate radicals and/or radial ions [21]. These radicals promote the formation of disulfide bonds leading to protein multimerization [20] and generate other reaction products [4]. Thiyl radicals produced from *N*-Hcy-protein undergo hydrogen atom transfer reactions, which generate C*^α^*-centered radicals, well-known precursors of protein carbonyls [4, 21], which can account for an increased susceptibility of *N*-Hcy-proteins to oxidative damage [2]. These processes can also account for the findings of the present work showing that increasing *N*-Hcy-keratin levels in mouse hair leads to a progressive loss of its solubility.

To substantiate a conclusion that *N*-homocysteinylation causes keratin damage, we used mouse models of HHcy in which *N*-homocysteinylation is induced by the inactivation of the *Cse* (the present work, Table 3), *Cbs*, and *Mthfr* genes [2]. These mice have 3- to12-fold higher *N*-Hcy-keratin levels than the wild-type animals. Our findings that the solubility of *N*-Hcy-keratin in SDS is inversely correlated with the *N*-Hcy-keratin content in these mice suggests that the increasing extent of *N*-homocysteinylation causes progressive keratin damage. This damage is also reflected by significantly reduced solubility of total keratin from hair of *Cbs*^−/−^ mice compared to their *Cbs*^+/+^ littermates (Table 4).

The *Cbs*^−/−^ mice have sparse hair with a significantly smaller diameter, compared to the *Cbs*^+/+^ animals [14, 22]. It is likely that this morphological hair defect could be caused, at least in part, by the physicochemical impairments of keratin structure caused by greatly increased *N*-homocysteinylation in *Cbs*^−/−^ mice, which is reflected by its reduced solubility in SDS (Tables 3 and 4).

Assuming that 1 mg hair contains about 20 nmol keratin, the 32% loss of solubility of total keratin (Table 4) corresponds to 6.4 nmol/mg hair more of SDS-insoluble keratin in *Cbs*^−/−^ mice relative to their *Cbs*^+/+^ littermates. This value is 6-fold greater than the *N*-Hcy-keratin content in *Cbs*^−/−^ mouse hair (1014 ± 86 pmol/mg hair, Table 3), suggesting that the aggregated *N*-Hcy-keratin (Table 3) serves as a “seed” that causes aggregation and a loss of solubility of unmodified keratin. These findings strongly suggest that the mechanism of protein aggregation induced by *N*-homocysteinylation demonstrated *in vitro* for the aggregation of albumin in the presence of a small amount of *N*-Hcy-albumin “seed” [8] also occurs *in vivo* in the mouse hair.

Protein damage can also be induced by *S*-thiolation, as recently shown for human serum albumin from hyperlipidemia patients, which carries Hcy and Cys bound via a disulfide bond to albumin Cys residues normally engaged in intrachain disulfide bonds [23]. It cannot be excluded that keratin damage can also be caused by the disruption of keratin disulfide bonds by *S*-homocysteinylation. However, this has not been studied in the present work, because *S*-Hcy-keratin could not be separated from Hcy-keratin.

About three dozens of individual Hcy-proteins have been identified in humans and animals, including albumin, fibrinogen [2], collagen [7], dynein [5], actin and E-cadherin [6], and major urinary protein [17]. The present study adds mammalian and bird keratins to the list of Hcy-proteins identified *in vivo*.

In conclusion, our findings show that hair keratin in animals and birds is a target for homocysteinylation *in vivo* and that this process causes keratin damage, manifested by the reduction of its solubility.

## Figures and Tables

**Table 1:** Hcy- and *S*-Hcy-keratin levels and solubility in animal hair.

Animal (*n*)	Hcy-keratin (pmol/mg hair)	*S*-Hcy-keratin (pmol/mg hair)	Hcy-keratin/(Hcy-keratin + *S*-Hcy-keratin)	SDS-insoluble Hcy-keratin fraction
Cat (5)	278 ± 157	422 ± 214	0.60 ± 0.16	0.70 ± 0.05
Cow (5)	556 ± 552	112 ± 54	0.70 ± 0.28	0.87 ± 0.11
Chipmunk (3)	200 ± 193	395 ± 189	0.56 ± 0.04	0.73 ± 0.01
Dog (3)	430 ± 80	386 ± 456	0.62 ± 0.29	0.69 ± 0.08
Goat (2)	177 ± 127	217 ± 16	0.42 ± 17	0.80± 0.10
Guinea pig (1)	56	253	0.18	0.67
Horse, tail hair base (10)	189 ± 230	48 ± 40	0.30 ± 0.25	0.92 ± 0.05
Horse, tail hair tip 60 cm (10)	1733 ± 882	31 ± 5	0.96 ± 0.01	1.00 ± 0.00
Mammoth (3)	688 ± 72	49 ± 20	0.93 ± 0.03	0.67 ± 0.20
Opossum (2)	484 ± 147			
Pig (2)	210 ± 206	131 ± 117	0.60 ± 0.04	0.67 ± 0.12
Rabbit (3)	146 ± 75	265 ± 59	0.35 ± 0.16	0.76 ± 0.03
Raccoon (3)	147 ± 58	212 ± 50	0.40 ± 0.04	0.66 ± 0.04
Sheep (3)	310 ± 15	271 ± 36	0.53 ± 0.02	0.67 ± 0.19
Squirrel (3)	352 ± 433			
Woodchuck (3)	733 ± 483	531 ± 474	0.57 ± 0.16	0.75 ± 0.09
Robin (4)	203 ± 134	76	0.80	0.92
Raven (1)	246	62	0.80	0.93
Turkey vulture (1)	125	72	0.63	0.81
Pigeon (1)	716	87	0.89	0.91
Seagull (1)	493	114	0.81	0.78

**Table 2:** Comparison of Hcy- and *S*-Hcy-keratin levels and solubility in mammal and bird pelage.

Animals (number of species, *n*)	Hcy-keratin (pmol/mg hair)	*S*-Hcy-keratin (pmol/mg hair)	Hcy-keratin/(Hcy-keratin + *S*-Hcy-keratin)	SDS-insoluble Hcy-keratin fraction
Mammals (12)	277 ± 202	247 ± 146	0.47 ± 0.15	0.75 ± 0.12
Birds (5)	556 ± 552	112 ± 54	0.79 ± 0.10	0.87 ± 0.07
*t*-test	0.518	0.037	0.0003	0.019

**Table 3:** Hcy-keratin and *S*-Hcy-keratin levels and the solubility of hair Hcy-keratin in HHcy and control wild-type mice.

Genotype (*n*)	Hcy-keratin (pmol/mg hair)	*S*-Hcy-keratin (pmol/mg hair)	Hcy-keratin/(Hcy-keratin + *S*-Hcy-keratin)	SDS-soluble Hcy-keratin fraction
*Cbs* ^−/−^ (4)	1014 ± 86^∗^	1937 ± 336^∗^	0.35 ± 0.03	0.07 ± 0.03^∗^
*Cse* ^−/−^ (5)	546 ± 46^∗^	682 ± 144^∗^	0.55 ± 0.04	0.14 ± 0.01^∗^
*Mthfr* ^−/−^ (7)	302 ± 71^#^	248 ± 121^#^	0.57 ± 0.17	0.19 ± 0.03^#^
Wild type + Met (6)	97 ± 11	303 ± 58^∗^	0.24 ± 0.02	0.31 ± 0.07
Wild type (28)	87 ± 10	127 ± 10	0.41 ± 0.21	0.36 ± 0.09

^∗,#^Significantly different from wild type; ^∗^*P* < 0.001,^#^*P* < 0.05.

**Table 4:** Effect of mouse *Cbs* genotype on the solubility of total hair keratin.

Genotype (*n*)	Total SDS-soluble keratin, relative values (*n*)	
	Hair stored at −80°C	Hair stored at 25°C, 6 months
*Cbs* ^−/−^ (5–8)	1.17 ± 0.11 (8)	1.51 ± 0.14 (5)
*Cbs* ^+/+^ (5–8)	1.18 ± 0.11 (8)	2.22 ± 0.05 (5)
*P* value	0.842	4.2 × 10^−6^

**Table 5:** Levels of copper, iron, and Met-keratin in *Cse*^−/−^ and *Cse*^+/+^ mouse pelage hair.

Genotype (*n*)	Copper (*μ*g/g hair)	Iron (*μ*g/g hair)	Met-keratin (nmol/mg hair)
*Cse* ^−/−^ (10)	4.83 ± 0.70	34.6 ± 10.7	41.0 ± 10.2
*Cse* ^+/+^ (12)	5.37 ± 1.43	40.1 ± 16.0	38.8 ± 6.4
*P* value	0.296	0.373	0.571

## Data Availability

The authors declare that all data used to support the findings of this study are included within the article.

## Abbreviations

DTT: Dithiothreitol
Cbs: Cystathionine *β*-synthase
Cse: Cystathionine *γ*-lyase
Mthfr: Methyltetrahydrofolate reductase
Cys: Cysteine
Hcy: Homocysteine
HHcy: Hyperhomocysteinemia
HPLC: High-performance liquid chromatography
Hcy-protein: Hcy linked to a protein via an amide (peptide or isopeptide) bond
Met: Methionine
NAC: *N*-acetyl cysteine
*N*-Hcy-protein: Hcy linked to a protein via an isopeptide bond
OPA: *o*-Phthaldialdehyde
*S*-Hcy-protein: Hcy linked to a protein via a disulfide bond
SDS: Sodium dodecyl sulfate
TCEP: Tris(2-carboxyethyl)phosphine.
